# Spontaneous Cure of an Ovarian Tumour

**Published:** 1848-10

**Authors:** 


*Spontaneous Cure of an Ovarian Tumour.*—In one of the sittings of the Société Medico-pratique of Paris, a case of encysted ovarian tumour, of several years standing, was brought forward, which disappeared in a few days, after very considerable micturition. M. Dobigney, who attended the lady, (of middle age,) asked the Society for the solution of the problem, whether the cyst opened into the bladder, or was merely effused into the peritoneum, absorbed and carried off by the kidneys. He gives no other symptom but a feeling, expressed by the patient, as if some liquid were falling drop by drop into the cavity of the abdomen. Another member mentioned a similar case which had occurred in his practice. We do not find that any of the members present gave an explanation of the phenomena, nor was it very easy to do so, considering the imperfect account of symptoms given.—*Med. Times.*

